# Socioeconomic inequalities in employees’ health-enhancing physical activity: Evidence from the SHAHWAR cohort study in Iran

**DOI:** 10.1371/journal.pone.0285620

**Published:** 2023-05-15

**Authors:** Maryam Khoramrooz, Fariba Zare, Farideh Sadeghian, Ali Dadgari, Reza Chaman, Seyed Mohammad Mirrezaie

**Affiliations:** 1 Department of Health Management and Economics, School of Public Health, Hamadan University of Medical Sciences, Hamadan, Iran; 2 Department of Epidemiology, School of Public Health, Iran University of Medical Sciences, Tehran, Iran; 3 Center for Health Related Social and Behavioral Sciences Research, Shahroud University of Medical Sciences, Shahroud, Iran; 4 School of Nursing and Midwifery, Shahroud University of Medical Sciences, Shahroud, Iran; 5 Department of Epidemiology, School of Public Health, Shahroud University of Medical Sciences, Shahroud, Iran; University of Georgia, UNITED STATES

## Abstract

**Background:**

Increasing level of physical activity (PA) among working population is of particular importance, because of the high return of investment on employees’ PA. This study was aimed to investigate socioeconomic inequalities in Health-Enhancing Physical Activity (HEPA) among employees of a Medical Sciences University in Iran.

**Methods:**

Data were extracted from the SHAHWAR Cohort study in Iran. Concentration index (C) and Wagstaff decomposition techniques were applied to determine socioeconomic inequality in the study outcomes and its contributors, respectively.

**Results:**

Nearly half of the university employees (44.6%) had poor HEPA, and employees with high socioeconomic status (SES) suffered more from it (C = 0.109; 95% CI: 0.075, 0.143). Also, we found while poor work-related PA (C = 0.175; 95% CI: 0.142, 0.209) and poor transport-related PA (C = 0.081, 95% CI: 0.047, 0.115) were more concentrated among high-SES employees, low-SES employees more affected by the poor PA at leisure time (C = -0.180; 95% CI: -0.213, -0.146). Shift working, and having higher SES and subjective social status were the main factors that positively contributed to the measured inequality in employees’ poor HEPA by 33%, 31.7%, and 29%, respectively, whereas, having a married life had a negative contribution of -39.1%. The measured inequality in poor leisure-time PA was mainly attributable to SES, having a married life, urban residency, and female gender by 58.1%, 32.5%, 28.5%, and -32.6%, respectively. SES, urban residency, shift working, and female gender, with the contributions of 42%, 33.5%, 21.6%, and -17.3%, respectively, were the main contributors of poor work-related PA inequality. Urban residency, having a married life, SES, and subjective social status mainly contributed to the inequality of poor transport-related PA by 82.9%, -58.7%, 36.3%, and 33.5%, respectively, followed by using a personal car (12.3%) and female gender (11.3%).

**Conclusions:**

To reduce the measured inequalities in employees’ PA, workplace health promotion programs should aim to educate and support male, urban resident, high-SES, high-social-class, and non-shift work employees to increase their PA at workplace, and female, married, rural resident, and low-SES employees to increase their leisure-time PA. Active transportation can be promoted among female, married, urban resident, high-SES, and high-social-class employees and those use a personal car.

## Introduction

Insufficient physical activity (PA) is a leading risk factor for major non-communicable diseases [1]. In 2019, 15.74 million disability-adjusted life years and 0.83 million deaths in the world were attributable to physical inactivity [2]. The risk of death increased from 20% to 30% among individuals suffering from inadequate PA, compared to those who have a minimum of 30 minutes moderate PA most days of the week [3].

In recent years, World Health Organization (WHO) has identified PA as a worldwide public health problem, and has determined the goal of 10% decrease in physical inactivity for its members states by 2025 [4]. Iran is located in Middle East region of southwestern Asia and it is among the 21 members states of Eastern Mediterranean Region of the WHO. Low PA contributed to 1.2 million deaths globally and 18,000 deaths in Iran in 2017 [5]. A study in 30 provinces in Iran indicated that the prevalence of adults’ physical inactivity was 54.7% (women: 61.9%, and men: 45.3%). Work-related activities had the largest contribution to the total PA [6]. Another study showed that the prevalence of PA among Iranian population is 30–70%, depending on the people’s gender and age [7].

PA has several health benefits, including lower risk of heart disease, stroke, type 2 diabetes, breast cancer and colon cancer [1], better mental health [1, 8], and it is estimated to increase life expectancy [1]. In addition, it could increase the employees’ productivity [9, 10], reduce their sick leaves [10, 11], and healthcare costs [12] by creating a healthier workforce. The results of a study by Wang et al. indicated that healthcare costs for employees who were active and very active were about $250 less than those of employees with sedentary lifestyle [12].

PA can be performed in different forms such as walking, cycling, sports, active recreation, work-related and home-based activities [13]. It is categorized in four main domains of leisure-time PA, domestic and gardening activities, work-related PA, and transport-related PA [14]. Regular activities with sufficient duration and intensity can provide health benefits for people [13]. However, to improve individuals’ health, avoiding physical inactivity is not sufficient, and having a definite level of PA, known as the Health-Enhancing PA (HEPA) is necessary [14]. WHO recommends more than 300 minutes of moderate PA, or more than 150 minutes of vigorous PA, or some equal combinations of both per week to gain additional health benefits [3]. Furthermore, there are some shreds of evidence which indicate that some domains of PA have different health benefits [15, 16]. Scarabottolo et al. in their study found associations between domains of PA and some aspects of health-related quality of life (HRQoL); The PA in the work-related domain was inversely related to functional capacity, the PA over sports in leisure time was positively related to vitality and mental health. The PA in leisure time and locomotion were contrariwise related to functional capacity, and positively associated with vitality and mental health. The overall PA was contrariwise associated with the functional capacity and positively related to body pain, vitality, and mental health [17].

The global studies indicated that several demographic and socioeconomic factors such as age, gender, education, occupation, income and mental health are associated with PA as a whole and four domains of it [8, 18–20]. A study conducted by Lim et al. among working women in Singapore during the COVID-19 outbreak showed of 217 participants, 32.7% achieved a HEPA level, whereas 44.7% were in sedentary state for 7 hours or more daily. The HEPA level were significantly associated with average daily sitting hours, occupation, income, and ethnicity [21]. Furthermore, there are different patterns for the impact of socioeconomic status (SES) on PA at different domains [22]. Governments often implement health promotion programs to promote public health. In this regard, the community dwellers’ workplaces are the appropriate settings for implementation of these programs [23, 24], since they spend a significant proportion of their time at work [22]. Moreover, due to its favorable effects on productivity, reducing absenteeism, and healthcare costs, the return of investment on employees’ PA is high [25]. Designing equitable interventions requires analyzing the current situation of PA distribution among different socioeconomic groups of employees and understanding its contributing factors. In Iran, some studies have been conducted on the impact of SES on PA in the general population. However, the employees’ PA has not been the focus of these studies [26–28]. Employees of medical sciences universities in Iran include a wide range of occupational disciplines, as the officer (accountants, IT experts, computer operators, etc.), medical staffs (physicians, nurses, nurse assistants, radiologists, clinical laboratory staffs, and technicians), and technical and service workers. Therefore, this study was designed to provide further evidence to address socioeconomic inequalities in employees’ PA, with special emphasis on the HEPA, among employees of a university of medical sciences in northeast of Iran.

## Materials & methods

### Source of data

The study data were extracted from the first phase of the SHAHWAR (SHAhroud Healthcare Workers Associated Research) Cohort study which was conducted from October 2, 2019 to September 21, 2020 in Shahroud, located in the northeast of Iran. SHAHWAR is a subset of PERSIAN Cohort study and focuses on the health of employees [29]. In this cohort study, data from 1178 personnel of Shahroud Medical University (SHMU) was prospectively collected after obtaining written informed consent. After cleaning data and excluding subjects with missing observations, a total of 1151 employees were included in the study analyses. The SHAHWAR Cohort study was approved by the Ethics Committee of Shahroud University of Medical Sciences (IR.SHMU.REC.1397.033).

The study participants’ PA was measured using the International PA Questionnaire (IPAQ), a valid and reliable questionnaire that was used to assess the participants’ PA in the PERSIAN Cohort study [30]. The questionnaire measures the amount of PA in the last 7 days in four domains: a) leisure-time PA, including all physical activities that respondent did exclusively for recreation, sport, exercise or leisure, b) domestic and gardening (yard) activities, including physical activities that respondent have carried out in and around her/his home, like housework, gardening, yard work, general maintenance work, and caring for his/her family, c) work-related PA, including paid jobs, farming, volunteer work, course work, and any other unpaid work that respondent did outside her/his home, and d) transport-related PA, including questions about how the respondent traveled from place to place, including workplaces, stores, movies, and so on [31]. According to the IPAQ standard protocol, PA in each domain was converted into the metabolic equivalent rates (METs) which were summed up to calculate the total PA [32].

### Variables definition

The outcome variables were poor HEPA and poor PA in four domains, as poor leisure-time PA, poor domestic and gardening activities, poor work-related PA, and poor transport-related PA. Participants were categorized into the three levels of PA as inactive, minimally active and active (having health-enhancing PA), according the protocol provided by IPAQ [14]. Employees who were inactive or minimally active were defined as who had poor HEPA. Furthermore, participants were categorized as having poor PA in each domain, if their activity was less than the median level of PA in that domain.

The Principal Component Analysis (PCA) was used to construct an index of SES for the study participants [33]. SES index is derived from a factor analysis of preliminary variables that included: household’s assets and properties, housing characteristics, entertainment and travel related variables, education and access to information, and job categories, including technical and service jobs vs. medical and office jobs. SES scores were used to classify participants into the five quintiles from the lowest (1^st^ quintile) to the highest (5^th^ quintile).

The study explanatory variables were as follows: demographic variables (gender, age, household size, and marital status), socioeconomic variables (place of residence, SES, subjective social status, and using a personal car), and work-related variables (having secondary job and shift working). The variable subjective social status was defined as the employees’ self-reported social class; We asked the respondents to answer the question "If the society in which you currently live is divided into 5 classes in terms of socioeconomic status, in which class is your family?" with 5 answers as "low", "middle-low", "middle", "middle-high", and "high". Due to a low frequency of the study participants in some groups, a new classification (low, middle, and high) was used in the study analyses.

### Inequality measurement

In this study, we used the familiar concentration index (C) approach [34] to measure socioeconomic inequalities in employees’ poor PA. The value of C ranges from –1 to +1. The C could take a negative (positive) value, indicating that poor PA was more concentrated among low- (high-) SES employees. When the C equals zero, it implies that poor PA was equally distributed among employees from different socioeconomic groups. The conventional Cs of the poor HEPA and in four domains of PA were calculated as follows:

$$c=\frac{2}{n\mu }\sum_{i=1}^{n}y_{i}r_{i}-1$$ (1)

In the equation above, *c* is the conventional C, *y*_*i*_ is poor PA of *i*^*th*^ employee, *r*_*i*_ is the fractional rank of *i*^*th*^ employee in the distribution of their SES, and μ is the mean of poor PA. Since the outcome variables were binary, we used the Wagstaff approach [35] to normalize the conventional Cs of poor PA using the formula below:

$$C=\frac{c}{\left(1-\mu \right)}$$ (2)

Where *C* is the Wagstaff normalized C.

### Decomposition of inequality

As it is shown by Wagstaff et al [36], measured inequality in health outcomes can be decomposed to the sum of contributions of its associated factors (the explained component) and an unexplained residual component. In the present study, we used this approach to quantify the contribution of the study explanatory variables to the measured inequalities in poor PA using the formula below:

$$C=\sum_{k}\left(\frac{\beta_{k}{\bar{X}}_{k}}{\mu }\right)C_{k}+\frac{C_{e}}{\mu }$$ (3)

Where *β*_*k*_ is the marginal effect of the *k*^*th*^ explanatory variable on the poor PA (estimated using the logit regression model), ${\bar{X}}_{K}$ is the mean of explanatory variables and μ is the mean of poor PA. The first component of the *C* is the sum of absolute contributions of the explanatory variables to the measured C which was calculated through multiplying the elasticity of poor PA with respective to the explanatory variables ($\frac{\beta_{k}{\bar{X}}_{k}}{\mu }$) by their Cs (*C*_*k*_). The *C*_*k*_ shows inequality in the distribution of the *k*^*th*^ explanatory variable among different-SES employees, and it was estimated similar to the Cs of the study outcomes. The residual component ($\frac{C_{e}}{\mu }$) is part of the measured inequality in poor PA that has not been explained by the study explanatory variables, and it was calculated as the C of outcome variable minus the sum of absolute contribution of the study explanatory variables.

All the study analyses were performed using the Stata software version 14.

## Results

Employees whose data were used in the study analyses consisted of 311 (27.02%) office staffs, 614 (53.34%) medical workers, and 226 (19.64%) technical and service workers. As it is shown in Table 1, among the study participants, 59.7% were female, most of them were in the age groups of 30–39 and 40–49 years (46.1% and 32.8%, respectively), the household size of 72.4% of participants was 3–4, most of them were married (87.5%), urban residents (90.4%), and had no secondary job (82.7%), and, more than half of them were from the middle social class, used their personal car, and were non-shift-worker (57.2%, 54.6%, and 58.2%, respectively).

**Table 1:** Descriptive statistics for employees in total and by all types of poor PA.

Characteristics	Total N (%)	PHEPA N (%)	PLTPA N (%)	PDGA N (%)	PWRPA N (%)	PTRPA N (%)
**Demographic characteristics**						
Gender						
Male	464 (40.31)	205 (44.18)	179 (38.58)	274 (59.05)	246 (52.56)	210 (45.26)
Female	687 (59.69)	308 (44.83)	393 (57.21)	242 (35.23)	242 (52.16)	350 (50.95)
*P-value*	*-*	*0*.*827*	*<0*.*001*	*<0*.*001*	*0*.*156*	*0*.*058*
Age (Years)						
20–29	107 (9.30)	39 (36.45)	56 (52.34)	67 (62.67)	39 (36.45)	56 (52.34)
30–39	531 (46.13)	228 (42.94)	272 (51.22)	207 (38.98)	257 (48.40)	276 (51.98)
40–49	377 (32.75)	178 (47.21)	177 (46.95)	172 (45.62)	202 (53.58)	173 (45.89)
≥50	136 (11.82)	68 (50.00)	67 (49.26)	70 (51.47)	73 (53.68)	55 (40.44)
*P-value*	*-*	*0*.*106*	*0*.*585*	*<0*.*001*	*0*.*012*	*0*.*051*
Household size						
≤2	184 (15.99)	81 (44.02)	87 (47.28)	105 (57.07)	94 (51.09)	88 (47.83)
3–4	845 (72.41)	385 (45.56)	421 (49.82)	352 (41.66)	422 (49.94)	413 (48.88)
≥5	122 (10.60)	47 (48.52)	64 (52.46)	59 (48.36)	55 (45.08)	59 (48.36)
*P-value*	*-*	*0*.*339*	*0*.*668*	*0*.*001*	*0*.*549*	*0*.*965*
Marital status						
Without spouse	144 (12.51)	49 (34.03)	54 (37.50)	83 (57.64)	66 (45.83)	55 (38.19)
Married	1007 (87.49)	464 (46.08)	518 (51.44)	433 (43.00)	505 (50.15)	505 (50.15)
*P-value*	*-*	*0*.*007*	*0*.*002*	*0*.*001*	*0*.*333*	*0*.*007*
**Socioeconomic characteristics**						
Place of residence						
Rural	111 (9.64)	52 (46.85)	65 (58.56)	44 (39.64)	49 (44.14)	42 (37.84)
Urban	1040 (90.36)	461 (44.33)	507 (48.75)	472 (45.38)	522 (50.19)	518 (49.81)
*P-value*	*-*	*0*.*612*	*0*.*049*	*0*.*247*	*0*.*226*	*0*.*016*
Socioeconomic status						
1^st^ quintile (lowest)	233 (20.24)	82 (35.19)	129 (55.36)	117 (50.21)	86 (36.91)	91 (39.06)
2^nd^ quintile	229 (19.90)	106 (46.29)	126 (55.02)	104 (45.41)	109 (47.60)	111 (48.47)
3^rd^ quintile	231 (20.07)	101 (43.72)	132 (57.14)	84 (36.36)	117 (50.65)	123 (53.25)
4^th^ quintile	229 (19.90)	110 (48.03)	106 (46.29)	107 (47.72)	121 (52.84)	122 (53.28)
5^th^ quintile (highest)	229 (19.90)	114 (49.78)	79 (34.50)	104 (45.41)	138 (60.26)	113 (49.34)
*P-value*	*-*	*0*.*015*	*<0*.*001*	*0*.*044*	*<0*.*001*	*0*.*014*
Subjective social status						
Low	76 (6.60)	26 (34.21)	33 (43.42)	38 (50.00)	30 (39.47)	32 (42.11)
Middle	658 (57.17)	278 (42.25)	344 (52.28)	297 (45.14)	312 (47.42)	300 (45.59)
High	417 (36.23)	209 (50.12)	195 (46.76)	181 (43.41)	229 (54.92)	228 (54.68)
*P-value*	*-*	*0*.*007*	*0*.*111*	*0*.*552*	*0*.*011*	*0*.*007*
Using a personal car						
No	523 (45.44)	231 (44.17)	286 (54.68)	229 (43.79)	259 (49.52)	289 (54.94)
Yes	628 (54.56)	346 (55.10)	286 (45.54)	287 (45.70)	312 (49.68)	239 (45.70)
*P-value*	*-*	*0*.*802*	*0*.*002*	*0*.*515*	*0*.*957*	*0*.*067*
**Work-related characteristics**						
Have a secondary job						
No	952 (82.71)	430 (45.17)	497 (52.21)	424 (44.54)	471 (49.45)	479 (50.32)
Yes	199 (17.29)	83 (41.71)	75 (37.69)	92 (46.23)	100 (50.25)	81 (40.70)
*P-value*	*-*	*0*.*372*	*<0*.*001*	*0*.*662*	*0*.*842*	*0*.*014*
Shift worker						
No	670 (58.21)	365 (54.48)	315 (47.16)	292 (43.58)	411 (61.34)	314 (46.87)
Yes	481 (41.79)	171 (35.33)	256 (53.22)	224 (46.57	160 (33.26)	246 (51.14)
*P-value*	*-*	*<0*.*001*	*0*.*043*	*0*.*315*	*<0*.*001*	*0*.*152*

^**†**^Abbreviations; PA: Physical activity, PHEPA: Poor health-enhancing PA, PLTPA: Poor leisure-time PA, PDGA: Poor domestic and gardening activities, PWRPA: Poor work-related PA, PTRPA: Poor transport-related PA

The results of our study also indicated 513 (44.6%) of the university employees had poor HEPA [432 (37.5%) and 81 (7.1%) of employees were minimally active and inactive, respectively]. According to the study results in Table 1, poor HEPA was more prevalent among employees who were married, with higher SES and social class, and non-shift-workers (P<0.05). The prevalence of poor leisure-time PA among female and married employees, and those who reside in rural areas, had lower SES, no personal car, and no secondary job, and shift-workers was more than their counterparts (P<0.05). Poor domestic and gardening activities was more prevalent among the male and 20–29 years old employees, and those who had the household size of ≤2, were without spouse, and were in 1^st^ and 4^th^ SES quintiles (P<0.05). Employees with higher ages, higher SES and social class, and non-shift-workers had higher percentage of the poor work-related PA (P<0.05). Also, poor transport-related PA was more prevalent among married employees and those who reside in urban areas, were from higher social classes and in 3^rd^ and 4^th^ SES quintiles, and had no secondary job (P<0.05).

**Fig 1:**
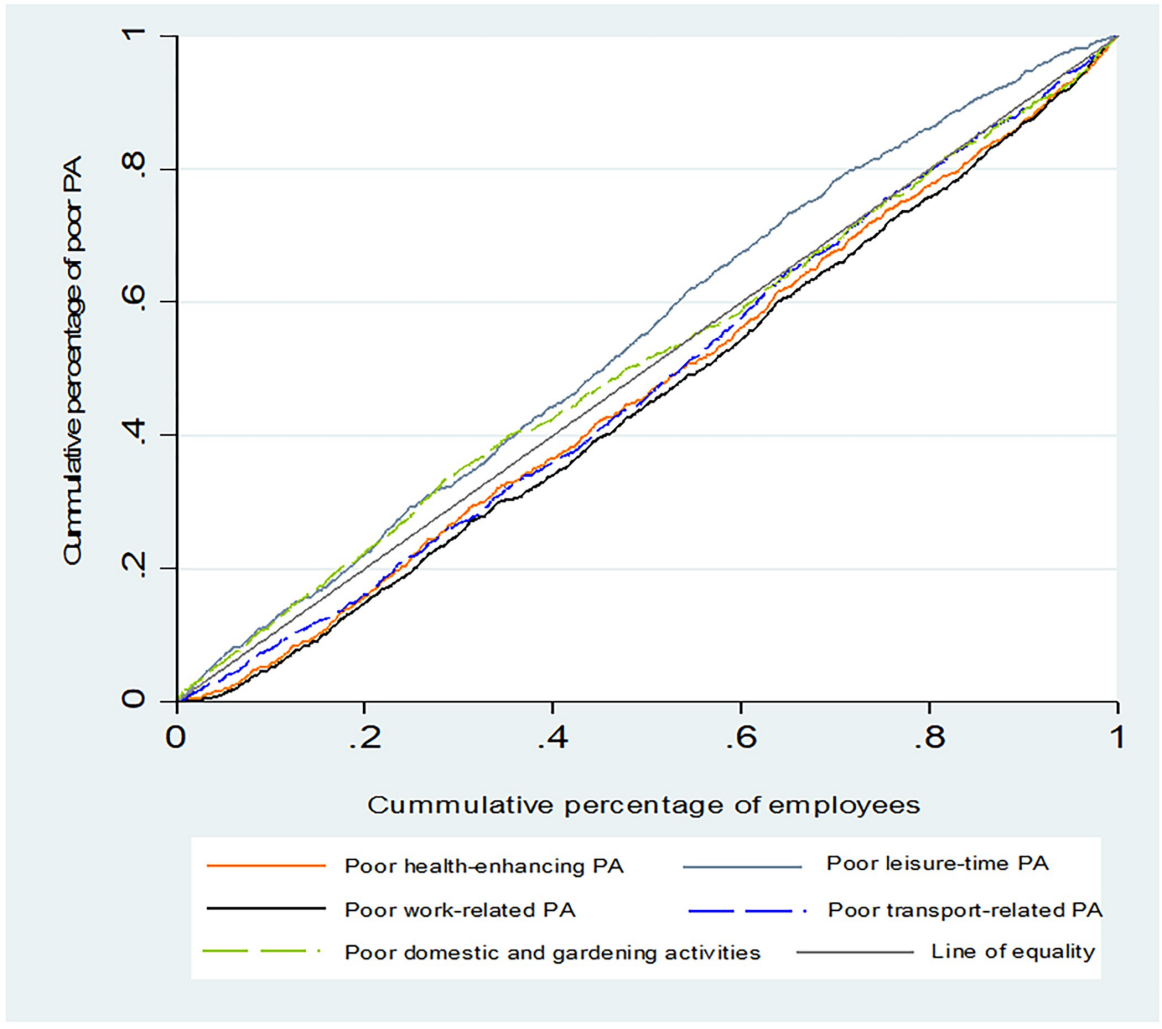
Concentration curves of all types of poor PA among employees.

The Cs and concentration curves of all types of poor PA are presented in Table 2 and Fig 1, respectively. The positive and statistically significant Cs of poor HEPA (C = 0.109; 95% CI: 0.075, 0.143), poor work-related PA (C = 0.175; 95% CI: 0.142, 0.209), and poor transport-related PA (C = 0.081, 95% CI: 0.047, 0.115), and also, lying their concentration curves above the line of equality show that they were more concentrated among high-SES employees. However, The C of poor leisure-time PA was negative and statistically significant (C = -0.180; 95% CI: -0.213, -0.146) and its concentration curve lies below the line of equality, indicating its more concentration among low-SES employees. The C of poor domestic and gardening activities was not statistically different from zero (C = 0.035; 95% CI: 0.001, 0.069) and its concentration curve crosses the line of equality, suggesting no socioeconomic inequality in its distribution among employees from the five socioeconomic groups.

**Table 2:** Concentration indices for all types of poor PA among employees.

Types of poor PA	Concentration index	Robust standard error	P-value
PHEPA	0.109	0.034	0.002
PLTPA	-0.180	0.034	<0.001
PDGPA	-0.035	0.034	0.306
PWRPA	0.175	0.034	<0.001
PTRPA	0.081	0.034	0.017

^**†**^Abbreviations; PA: Physical activity, PHEPA: Poor health-enhancing PA, PLTPA: Poor leisure-time PA, PDGA: Poor domestic and gardening activities, PWRPA: Poor work-related PA, PTRPA: Poor transport-related PA

The results of multiple logistic regression analyses for the study outcomes were indicated in Table 3. Shift-workers were less likely to have poor health-enhancing PA, whereas, married employees were more probable to have poor health-enhancing PA. Furthermore, poor health-enhancing PA was less prevalent among employees from middle social class compared to those from high social class (P<0.05). When looking at poor leisure-time PA, it was more probable among female and married employees. However, employees in 4^th^ and 5^th^ SES quintiles were less likely to have poor leisure-time PA (P<0.05). The odds of poor domestic and gardening PA among females and married employees, and those with the age of 30–39 and 40–49 years, and the household size of 3–4 was less than their counterparts, whereas, it was more probable among urban residents (P<0.05). Females and shift-workers were less likely to have work-related PA, whereas, it was more probable among employees with the age of 40–49 years and those from higher SES quintiles (P<0.05). The odds of poor transport-related PA among married employees, and those use a personal car was more than other employees. Also, employees from middle social class and had a secondary job were less likely to have poor transport-related PA (P<0.05).

**Table 3:** Association of poor PA with the employees’ characteristics.

Characteristics	Adjusted odds ratios (95% CI)				
	PHEPA N (%)	PLTPA N (%)	PDGA N (%)	PWRPA N (%)	PTRPA N (%)
**Demographic characteristics**					
Female gender	0.88 (0.66, 1.16)	2.53 (1.90, 3.36)*	0.31 (0.23, 0.41)*	0.61 (0.46, 0.82)*	1.14 (0.87, 1.50)
Age (RC: 20–29)					
30–39	1.18 (0.74, 1.87)	0.86 (0.54, 1.35)	0.42 (0.26, 0.67)*	1.51 (0.95, 2.40)	0.90 (0.58, 1.41)
40–49	1.31 (0.80, 2.15)	0.74 (0.46, 1.21)	0.54 (0.33, 0.90)*	1.69 (1.03, 2.78)*	0.71 (0.44, 1.14)
≥50	1.46 (0.83, 2.59)	0.98 (0.55, 1.73)	0.57 (0.2, 1.02)	1.58 (0.89, 2.82)	0.67 (0.38, 1.16)
Household size (RC: ≤2)					
3–4	0.92 (0.65, 1.31)	0.86 (0.61, 1.24)	0.60 (0.42, 0.86)*	0.88 (0.62, 1.27)	0.95 (0.67, 1.34)
≥5	0.68 (0.40, 1.15)	1.00 (0.59, 1.68)	0.67 (0.40, 1.12)	0.74 (0.44, 1.25)	1.14 (0.69, 1.89)
Married	1.61 (1.08, 2.39)*	2.08 (1.40, 3.08)*	0.51 (0.34, 0.75)*	1.09 (0.74, 1.61)	1.74 (1.19, 2.55)*
**Socioeconomic characteristics**					
Urban residency	1.02 (0.66, 1.58)	0.73 (0.47, 1.14)	1.70 (1.09, 2.68)*	1.44 (0.93, 2.24)	1.47 (0.95, 2.27)
Objective SES (RC: 1^st^ quintile)					
2^nd^ quintile	1.43 (0.96, 2.13)	0.85 (0.57, 1.26)	1.02 (0.69, 1.52)	1.46 (0.98, 2.18)	1.28 (0.87, 1.88)
3^rd^ quintile	1.27 (0.84, 1.92)	0.84 (0.56, 1.26)	0.74 (0.49, 1.11)	1.70 (1.13, 2.56)*	1.47 (0.99, 2.19)
4^th^ quintile	1.46 (0.95, 2.23)	0.59 (0.39, 0.90)*	1.00 (0.65, 1.52)	1.74 (1.13, 2.66)*	1.45 (0.96, 2.20)
5^th^ quintile (highest)	1.38 (0.88, 2.16)	0.38 (0.24, 0.60)*	0.88 (0.56, 1.38)	2.08 (1.32, 3.28)*	1.26 (0.82, 1.95)
Subjective social status (RC: High)					
Middle	0.75 (0.57, 0.99)*	1.15 (0.87, 1.52)	0.88 (0.67, 1.17)	0.80 (0.60, 1.05)	0.75 (0.58, 0.99)*
Low	0.61 (0.35, 1.06)	0.93 (0.54, 1.61)	0.73 (0.42, 1.27)	0.58 (0.34, 1.02)	0.79 (0.46, 1.34)
Using a personal car	0.93 (0.72, 1.20)	0.92 (0.71, 1.19)	0.86 (0.66, 1.12)	0.80 (0.62, 1.04)	1.29 (1.01, 1.66)*
**Work-related characteristics**					
Shift worker	0.39 (0.30, 0.51)*	1.21 (0.93, 1.58)	1.01 (0.77, 1.32)	0.33 (0.25, 0.43)*	1.13 (0.88, 1.46)
Have a secondary job	0.77 (0.55, 1.09)	0.78 (0.55, 1.09)	0.74 (0.53, 1.04)	0.83 (0.59, 1.17)	0.70 (0.50, 0.97)*

* P<0.05

^**†**^Abbreviations; RC: Reference Category, PA: Physical activity, PHEPA: Poor health-enhancing PA, PLTPA: Poor leisure-time PA, PDGA: Poor domestic and gardening activities, PWRPA: Poor work-related PA, PTRPA: Poor transport-related PA, CI: Confidence interval

Table 4. represents the results of decomposition analyses for the observed socioeconomic inequalities in the study outcomes. Shift working, and having higher SES and subjective social status were the main factors that positively contributed to the more concentration of poor HEPA among high-SES employees by 33%, 31.7%, and 29%, respectively, whereas, having a married life had a negative contribution of -39.1%.

**Table 4:** Decomposition of socioeconomic inequalities in poor PA among employees.

Characteristics	Marginal effect				Elasticity				C_k_	Absolute contribution to CI				% Contribution			
	PHEPA	PLTPA	PWRPA	PTRPA	PHEPA	PLTPA	PWRPA	PTRPA		PHEPA	PLTPA	PWRPA	PTRPA	PHEPA	PLTPA	PWRPA	PTRPA
**Demographic characteristics**																	
Female gender	-0.030	0.211	-0.109	0.032	-0.040	0.253	-0.131	0.040	0.231	-0.009	0.059	-0.030	0.009	-8.49	-32.62	-17.25	11.26
Age (RC: 20–29)																	
30–39	0.037	-0.035	0.092	-0.024	0.038	-0.032	0.086	-0.023	0.061	0.002	-0.002	0.005	-0.001	2.16	1.10	3.01	-1.73
40–49	0.061	-0.067	0.117	-0.082	0.045	-0.044	0.077	-0.055	-0.084	-0.004	0.004	-0.007	0.005	-3.49	-2.07	-3.72	5.71
≥50	0.087	-0.005	0.102	-0.097	0.023	-0.001	0.024	-0.024	-0.108	-0.002	0.128*10^−3^	-0.003	0.003	-2.28	-0.07	-1.49	3.12
Sum										-0.004	0.002	-0.004	0.006	-3.61	-1.04	-2.21	7.11
Household size (RC: ≤2)																	
3–4	-0.016	-0.033	-0.027	-0.013	-0.030	-0.049	-0.040	-0.020	-0.044	0.001	0.002	0.002	0.001	1.21	-1.18	1.00	1.06
≥5	-0.088	0.000	-0.066	0.031	-0.021	0.000	-0.014	0.007	-0.352	0.007	0.000	0.005	-0.002	6.75	-0.01	2.85	-2.94
Sum										0.009	0.002	0.007	-0.002	7.96	-1.20	3.86	-1.88
Married	0.109	0.167	0.019	0.133	0.213	0.293	0.034	0.239	-0.199	-0.043	-0.058	-0.007	-0.048	-39.09	32.53	-3.82	-58.66
**Socioeconomic characteristics**																	
Urban residency	0.005	-0.071	0.081	0.092	0.010	-0.129	0.148	0.170	0.396	0.004	-0.051	0.059	0.067	3.71	28.50	33.47	82.92
Objective SES (RC: 1^st^ quintile-lowest)																	
2^nd^ quintile	0.082	-0.036	0.085	0.058	0.036	-0.015	0.034	0.024	-0.495	-0.018	0.007	-0.017	-0.012	-16.58	-4.00	-9.62	-14.53
3^rd^ quintile	0.055	-0.039	0.118	0.093	0.025	-0.016	0.048	0.038	0.004	0.107*10^−3^	-0.068*10^−3^	0.207*10^−3^	0.166*10^−3^	0.10	0.04	0.12	0.20
4^th^ quintile	0.086	-0.119	0.123	0.089	0.038	-0.048	0.049	0.037	0.503	0.019	-0.024	0.025	0.018	17.78	13.38	14.15	22.62
5^th^ quintile (highest)	0.074	-0.219	0.163	0.056	0.033	-0.088	0.066	0.023	1.000	0.033	-0.088	0.066	0.023	30.44	48.66	37.38	28.00
Sum										0.035	-0.104	0.074	0.030	31.73	58.07	42.03	36.30
Subjective social status (RC: High)																	
Middle	-0.064	0.033	-0.051	-0.068	-0.083	0.037	-0.059	-0.080	-0.305	0.025	-0.011	0.018	0.024	23.20	6.36	10.26	29.90
Low	-0.113	-0.016	-0.120	-0.057	-0.017	-0.002	-0.016	-0.008	-0.376	0.006	0.001	0.006	0.003	5.80	-0.43	3.42	3.58
Sum										0.032	-0.011	0.024	0.027	29.00	5.93	13.67	33.49
Using a personal car	-0.016	-0.019	-0.050	0.061	-0.020	-0.021	-0.055	0.069	0.145	-0.003	-0.003	-0.008	0.010	-2.67	1.67	-4.53	12.32
**Work-related characteristics**																	
Shift worker	-0.213	0.044	-0.250	0.030	-0.200	0.037	-0.210	0.026	-0.180	0.036	-0.007	0.038	-0.005	33.01	3.71	21.55	-5.67
Have a secondary job	-0.059	-0.057	-0.041	-0.086	-0.023	-0.020	-0.014	-0.030	0.131	-0.003	-0.003	-0.002	-0.004	-2.75	1.46	-1.07	-4.92
**Total observed**										**0.053**	**-0.174**	**0.150**	**0.091**	**48.81**	**96.99**	**85.71**	**112.27**
**Residual**										**0.056**	**-0.005**	**0.025**	**-0.010**	**51.19**	**3.01**	**14.29**	**-12.27**
**Total**										**0.109**	**-0.180**	**0.175**	**0.081**	**100**	**100**	**100**	**100**

^**†**^Abbreviations; RC: Reference category, PA: Physical activity, PHEPA: Poor health-enhancing PA, PLTPA: Poor leisure-time PA, PDGA: Poor domestic and gardening activities, PWRPA: Poor work-related PA, PTRPA: Poor transport-related PA

Similarly, SES, having a married life and urban residency positively contributed to the more concentration of poor leisure-time PA among low-SES employees by 58.1%, 32.5%, and 28.5%, respectively. Also, female gender had the most negative contribution of -32.6% to this inequality. SES, urban residency, and shift working all positively contributed to the measured inequality in poor work-related PA (its more concentration among high-SES employees) by 42%, 33.5%, and 21.6%, respectively, while the opposite is true for female gender by the contribution of -17.3%. Urban residency, SES, and subjective social status were the three main factors that increased the concentration of poor transport-related PA among high-SES employees by 82.9%, 36.3%, and 33.5%, respectively, followed by using a personal car (12.3%) and female gender (11.3%). However, having a married life decreased the measured inequality in poor transport-related PA by -58.7%.

## Discussion

The present study was aimed to investigate socioeconomic inequalities in PA among the Iranian employees of medical sciences universities. The results of our study showed that 44.6% of employees had poor health-enhancing PA. Other studies reported lower levels of HEPA in their samples [37, 38]. It seems the observed differences resulted from different studied populations. In our study, all of samples were health sector workers, whereas in other studies the PA of general population (including, employed people, housekeepers, retired, and so on) was assessed.

The results of the inequality measurement revealed several issues: 1) high-SES employees tend to have more poor health-enhancing PA than their low-SES counterparts; 2) different domains of PA show different patterns of socioeconomic inequality; and 3) high-SES employees were more affected by the poor PA in their workplace and transportation, while low-SES employees were more suffered from the poor PA in their leisure time. These findings are similar to those found in other researches on the general population [39–41].

There seem to be reasons why despite more opportunities for the high-SES employees to be physically active in their leisure time and transportation (including walking and cycling) [40, 41], health-enhancing PA among them stay low; One reason is that the high opportunity cost of leisure-time PA and active transportation may reduce tendency of high-SES employees to increase their PA in these domains [42, 43]. Health care workers (HCWs), in comparison with the general population, have more time constraints, which could decrease their frequency of PA participation [42], and opportunity cost of activities outside the workplace for high-SES employees [43] can lead them to have a time trade-off between working and PA in other domains of activities [42]. In the study conducted by Humphreys and Ruseski it has been shown that employment has an opportunity cost for people’s PA in other domains of activity, such that employed individuals participate in sports activities approximately one hour less per week than the unemployed [43].

Although the payment to health sector employees in Iran is subject to the coordinated system of payment [44] to all employees, however, a part of the payments, such as fee-for-service payments and extra compensation for full-time faculty members, and also, earnings from employment in the private sector, depends on the quantitative and qualitative performance of the employees. So, high-SES employees due to the possibility of earning higher income via devoting more time to work, and also difficulty of their work in terms of time and job stress, are less willing to devote more time to other areas, including PA in leisure time and transportation.

Decomposition analysis allowed us to explore the contribution of determinant factors to the measured inequalities in employees’ PA, as follows:

### Demographic factors

Married employees, who were commonly from low-SES groups, were more likely to have poor health-enhancing, leisure-time, and transport-related PA. However, poor domestic and gardening activities was less probable among married employees. These findings are in line with the results of other studies [45–47]. More requirements of married life and associated economic problems to meet them could increase opportunity costs of PA outside the home for low-SES married employees.

Female gender was another demographic factor which explains a part of the measured inequalities in employees’ PA in their leisure time, workplace, and transportation. Other studies in Iran show that PA is significantly less common among women than men in general population [48, 49]. Similar finding was seen in Abu Saad et al’s study which indicated female healthcare workers were less physically active than males [18]. Our results show that most of the female participants were from high-SES groups, and, while they were more likely to have more poor PA in leisure time, they less suffered from poor PA at workplace and in performing home activities compared to their male counterparts. Poor active transportation was not different among female and male participants, however, female gender had a contribution of 11.3% to the measured inequality in active transportation. Lack of structure for opportunities within communities, cultural constraints, economic, social, and personal home expectations are the main factors that could restrict high-SES females’ PA in their leisure- time and transportation [50, 51].

### Socioeconomic factors

Our study indicated that SES has a substantial contribution to the measured inequalities in employees’ poor HEPA, and also, their poor PA in leisure time, workplace, and transportation. High-SES employees, due to their higher education, more work experience or a more stable job position, usually work in jobs that require less PA. So, they were more likely to have poor work-related PA. Similar results were seen in other studies [18, 52, 53]. Our study also suggested almost one-half of the measured inequality in poor active transportation is attributable to SES. Some studies have shown that walkable built environment has a greater impact on active transportation among high-SES individuals than their low-SES counterparts [54, 55]. Therefore, in addition to the opportunity costs of active transportation, unsuitable walkable environment could be another reason for lower active transportation of high-SES employees in our study.

Results of our study for poor leisure-time PA was quite different; low-SES employees were more probable to have poor PA in their leisure time compared to their high-SES counterparts. This finding is in line with the results of a systematic review conducted by Kirk MA and Rhodes [56]. Due to their higher demanded activity jobs, low-SES employees usually do most of their daily PAs at workplace [22]. Therefore, they have less energy for PA in their leisure time. In addition, many leisure time activities involve monetary costs that these individuals may not be able to afford [57, 58]. Additionally, low-SES employees may live in rural areas and urban neighborhoods in which access to recreational and other facilities, their quality, and social and cultural factors could decrease their tendency to engage in leisure-time PA compared to the high-SES groups. The role of these factors in individuals’ decisions on PA in their leisure time has been well documented in the literature [59–62].

Urban residency was the second socioeconomic factor that positively contributed to the measured inequalities in poor leisure-time, work-related, and transport-related PA. Urban resident employees due to their higher SES, usually have more access to the parks, recreation and sport facilities, which were cited as the facilitators of leisure-time PA in other studies [59, 60]. In addition, as it is showed in other studies, even though people have equal access to recreational and other facilities for PA, the extent to which they use these facilities is affected by the quality of them [62–64] and social and cultural factors inherent in their residential area [61–64] which could negatively affect the rural resident employees’ decisions related to their PA in leisure time in our study.

Furthermore, employees living in urban areas, have different occupations in the university, including office, medical, technical and service jobs. However, most of the medical university employees in rural areas are primary health employees (Behvarz), who work in health houses. The job of primary health employees is more physically demanded because it requires a lot of work outside the health houses in the village [65]. These results imply that employees who live in urban areas were less physically active in their workplace and transportation than their rural counterparts.

Subjective social status is another factor which positively contributed to the measured inequalities in poor HEPA, and poor PA in workplace and transportation. In our study, employees from lower social classes had less poor health-enhancing, work-related, and transport-related PA than those from high social classes. Frerichs et als’ in their study in four Asian countries found an association between subjective social status and people’s weekly or daily PA [66]. The results of other studies indicate that people’s social norms, values and beliefs play an important role in their health behaviors, including PA. [67, 68] In our study, employees’ perception of their social position seems to be effective in their PA; such that those who consider themselves to have a higher social standing had less PAs in the workplace, they also may believe that there are many requirements over the economic costs to have sport activities [69], and had less activities such as walking and cycling. Because they are generally from high-SES groups, they don’t have much opportunity to compensate for the low activity in the workplace and transportation through more activity in leisure time. In this way, the health-enhancing PA among these employees is generally less than others.

Using a personal car in comparison to the previous socioeconomic factors had a less contribution to the measured inequality in active transportation, indicating that use of personal car for transportation dose not vary much between high- and low-SES employees.

### Work-related factors

Working in shift-work jobs positively contributed to the more concentration of poor HEPA and work-related PA among high-SES employees. Non-shift workers were more likely to have poor work-related PA and poor HEPA than their shift worker counterparts. This finding is in line with the results of previous studies [70–72]. It seems that high-SES non-shift-workers, often have less physically demanded jobs, among medical, technical, and service occupations, and their PA in workplace was less than their shift work counterparts.

Individuals and HCWs in special, have a finite amount of time and resources to devote to work and leisure activities [42], which is visible in both high- and low-SES employees. On the other hand, they earn money from work and spend money and time for other domains of activity. In our study, it seems that the pattern of decision-making for allocating time and resources to PA at work, leisure time, and transportation is different among high- and low-SES employees. Since PA programs should be population-based and accessible to those who need more PA, targeted or tailored strategies to move high-SES employees away from sedentary behavior should focus on encouraging PA at workplace, and reducing the opportunity costs of PA in leisure time and transportation. Low-SES employees should be targeted for interventions that encourages PA at leisure time through reducing opportunity costs of leisure activities.

### Strength and limitations

This is the first study which investigated socioeconomic inequalities in poor HEPA and all of its domains among employees of medical sciences universities. However, this study is subject to some limitations; First, in decomposition analysis, 48.8% of the measured socioeconomic inequality in poor HEPA was explained by the study explanatory variables. However, presence of the main factors which have either positive or negative contributions to the poor HEPA inequality, provides sufficient evidence to reduce inequality in employees’ poor HEPA. Second, due to the cross-sectional design of our study, casual interpretations should be done with caution.

## Conclusion

The findings of the present study provide new evidence of the current status of inequalities in employees’ PA and its contributing factors which can be used to design targeted interventions to eliminate these inequalities in the future. To reduce the measured inequalities in employees’ PA, workplace health promotion programs should aim to educate and support male, urban resident, high-SES, high-social-class, and non-shift work employees to increase their PA at workplace, and female, married, rural resident, and low-SES employees to increase their leisure-time PA. Active transportation can be promoted among female, married, urban resident, high-SES, and high-social-class employees and those use a personal car.
